# Completion Rates for Ecological Momentary Assessments of Food Intake During Pregnancy and Post Partum: Descriptive Study

**DOI:** 10.2196/67081

**Published:** 2025-10-08

**Authors:** Sarah Annalise Sanders, Serwaa Omowale, Andrea Casas, Alexis Kiyanda, Abigail Smith Kosbie, Yu-Hsuan Lai, Meredith Wallace, Stephen Rathbun, Tiffany Gary-Webb, Esa Davis, Lora Burke, Dara Méndez

**Affiliations:** 1 Department of Epidemiology, University of Pittsburgh, 130 De Soto Street, Pittsburgh, PA, 15261, United States, 1 412-648-5664; 2 Department of Management, Policy, and Community Health, University of Texas Health Science Center at Houston, Houston, TX, United States; 3 Department of Environmental and Occupational Health, University of Pittsburgh, Pittsburgh, PA, United States; 4 Department of Behavioral & Community Health Sciences, University of Pittsburgh, Pittsburgh, PA, United States; 5 Department of Psychiatry, University of Pittsburgh, Pittsburgh, PA, United States; 6 Department of Epidemiology & Biostatistics, University of Georgia, Athens, GA, United States; 7 Family and Community Medicine, School of Medicine, University of Maryland, Baltimore, MD, United States; 8 School of Nursing, University of Pittsburgh, Pittsburgh, PA, United States

**Keywords:** dietary behavior, ecological momentary assessment, pregnancy, post partum, dietary, diet, dietary intake, survey, longitudinal study

## Abstract

**Background:**

The collection of dietary behavior data is crucial in childbearing populations. In addition to observed inequities in perinatal dietary intake and quality, burdensome assessment methods (eg, 24-h dietary recall) may limit research participation for some groups. Ecological momentary assessment (EMA) is associated with reduced recall bias and participant convenience, but there is a dearth of studies with diverse cohorts.

**Objective:**

Our aim is to describe participant completion of food intake items in EMA surveys, overall and across individual characteristics (eg, prepregnancy BMI).

**Methods:**

Using secondary EMA data from participants in a longitudinal study, we report average completion rates of survey items regarding dietary behavior (eg, number of meals eaten in a day) across individual demographic variables (eg, age) and combined strata (eg, race+age) during late pregnancy and throughout 12 months post partum.

**Results:**

In our analytic sample (N=310), the average completion rate was 52.4% (SD 27.8%) during pregnancy, rising to 59.1% (SD 22.0%) after giving birth. Participants who were older (>30 y), overweight before pregnancy, self-identified as White, working, or earning higher annual income (>US $50,000) had higher average completion rates than their counterparts. Examining combined strata, we found some variation in survey completion within racial groups. Black participants using a study phone had higher average completion rates during pregnancy and post partum, but this relationship was reversed for White participants.

**Conclusions:**

Our secondary analysis showed relatively stable engagement with EMA surveys in a childbearing cohort across 15 months. Increased completion rates among privileged groups (eg, White, higher income) may demonstrate the impact of socioeconomic advantages on individual health behaviors. Investigators should consider how intersections between race and other factors (eg, employment) may impact participation and data collection.

## Introduction

The collection of dietary behavior data is integral to understanding how diet and eating patterns shape health for individuals and communities [1,2]. In childbearing populations, studies show that dietary intake patterns during pregnancy are critical to the health of both the mother and infant [3]. Inadequate nutrition during pregnancy can increase the risk of cardiometabolic comorbidities (eg, hypertension disorders of pregnancy and gestational diabetes) during pregnancy and of low birthweight from fetal growth restriction and neurodevelopmental delays [4,5]. Additionally, research shows significant disparities in perinatal diet quality across social determinants of health (education, food security, income) [6-9], obliging studies to examine dietary behaviors across these strata. Racial inequities also exist, where Black birthing populations report higher food insecurity [6] and lower-quality diets [7-9] and increased prevalence of perinatal and postnatal comorbidities compared to White populations [10].

Commonly used dietary assessment methods include 24-hour dietary recalls, food frequency questionnaires, and food diaries, each presenting advantages and disadvantages in their administration and the quality of data collected [11]. The 24-hour dietary recall provides detailed information on dietary intake and has proven to be acceptable across diverse populations including pregnant individuals [12-15]. The major disadvantages of the 24-hour dietary recall measure are that it is prone to recall bias, it is limited by its reliance on an individual’s episodic memory, there is potential for intentional misreporting, and it requires the greatest amount of time to complete [11,13]. Food frequency questionnaires also rely on participants’ memory and lack detailed collection of specific food intake. Food diaries and the 24-hour dietary recall are similar in recording detailed food intake, increasing the burden on study participants and potentially influencing their eating behavior on the days being recorded [16].

Ecological momentary assessment (EMA) is a sampling method used to repeatedly collect information in “real time” [17] and in one’s natural setting; it has been used to understand eating behaviors and food intake, typically via digital devices (eg, smartphones) [18]. EMA reduces retrospective bias and promotes reliability and ecological validity. Compared to more time-intensive methods, EMA reduces the burden of reporting and often the time lapse between a behavior and its recording. Furthermore, the repeated sampling design facilitates a broader behavioral assessment over time [17-22]. Recent research using EMA to assess dietary behaviors has been conducted in different populations, including children [23,24], adolescents [25,26], mother-child dyads [27-29], individuals with eating disorders [30], and postbariatric surgery patients [31]. The few EMA studies examining dietary behavior in perinatal populations report homogenous samples and poor adherence to EMA completion, making a strong case for more demographically diverse cohorts in evaluating the usability of these assessment tools [32,33].

Since EMA is a novel approach to collecting dietary data, particularly over long periods of time (eg, >3 mo), it is critical to assess participant adherence to the EMA protocol for reporting food intake. It is important to note that EMA surveys are designed to be briefer, making them ideal for studying the context of eating behaviors, rather than nutritional content, which may be measured more precisely in recalls and diaries. Leveraging EMA data from a diverse childbearing cohort within a longitudinal, observational investigation (approximately 15 mo), this secondary analysis aimed to describe participant completion of dietary intake items in EMA surveys, overall and across individual characteristics (eg, prepregnancy BMI).

## Methods

### Study Design

This secondary analysis uses participant-reported data regarding dietary intake from the Postpartum Mothers Mobile Study (PMOMS), a longitudinal, observational investigation [34] designed to examine factors associated with racial disparities in postpartum weight retention and cardiometabolic health. Pregnant people were recruited between 18 and 32 weeks’ gestation and followed through 12 months post partum. Participants completed daily EMA surveys via smartphones, weighed themselves weekly using Bluetooth-enabled scales, and attended follow-up visits for anthropometric measurements and biospecimen collection. The study applied EMA methods to better understand participants’ experiences and exposures in their natural environment via real-time measurements of psychosocial (eg, stress, discrimination), behavioral (eg, eating behavior, consumption of foods with added sugars, physical activity), and contextual (eg, geographic location) factors.

Brief EMA surveys were administered at the beginning of day (BOD) and end of day (EOD), representing time-contingent responses, and at random times throughout the day (signal-contingent responses). Participants selected times for receiving BOD and EOD surveys, with at least 9 hours between the 2 surveys. Links for BOD and EOD surveys were active for 30 minutes (60 min for random surveys) to ensure that participant-reported data reflected real-time information. Each participant contributed approximately 15 months of EMA data during the pregnancy and postpartum periods. The protocol for PMOMS is described in more detail in a previous publication [34].

### Ethical Considerations

The study was approved by the institutional review board within the University of Pittsburgh Human Research Protection Office (#PRO16100117).

### Participants and Recruitment

PMOMS was an ancillary study to a randomized clinical trial, the Comparison of Two Screening Strategies for Gestational Diabetes (GDM^2^) [35,36], conducted at a women’s hospital in southwestern Pennsylvania. The clinical trial visits provided 2 in-person opportunities for our staff to approach and recruit potential participants and determine eligibility between 18 and 32 weeks’ gestation. Participants were recruited this way between December 2017 and March 2019. After the GDM^2^ study ended recruitment, PMOMS staff directly recruited participants from December 2019 to March 2020, when study activities were interrupted by the COVID-19 pandemic.

After providing their written, informed consent at a baseline visit (between 18 and 32 weeks’ gestation), participants completed demographic questionnaires and anthropometric measures. At this same visit, a research assistant facilitated setting up participants’ smartphones for receiving daily EMA surveys, which were scheduled to begin the following day [34]. Individuals using their own phone for study participation were compensated US $35 each month for using their data (up to US $525 throughout the study). For those without a suitable device for participating in the study, PMOMS offered a smartphone with a paid, unlimited data plan for the duration of the study. Upon completing the study, these participants had the option of keeping the smartphone provided by the study as an additional incentive.

All participants were compensated monthly if their EMA completion rate reached 60% or more during that month. Those who reached a completion rate of 80% or higher were entered into a monthly drawing for additional compensation. In addition, PMOMS staff conducted routine check-ins (via phone or text) with all participants falling below the 60% threshold to identify and address any barriers to increasing their completion rate. 

Pregnant individuals were excluded from participating in PMOMS if they indicated any of the following: plan to deliver at a different hospital from the study recruitment site, multiple pregnancy, plan to move from the area before 12 months post partum, history of type 1 or type 2 diabetes diagnosis, gestational diabetes diagnosis before 20 weeks’ gestation in current pregnancy, consumption of oral glucocorticoids in last 30 days, history of “dumping syndrome” following gastric bypass surgery, hypertension diagnosis or medication for high blood pressure, and diagnosis of severe liver disease (eg, cirrhosis). Further details about the PMOMS protocol can be found elsewhere [34].

### Measures

Although EOD surveys were delivered to participants every day, eating habits and intake of added-sugar foods were only assessed in these surveys on 10 randomly selected weekdays and 4 randomly selected weekend days for every 28-day block of time after EMA prompts began [34]. Participants were asked to report the number of meals and snacks they ate during the day. They were also asked to report whether they had any food or drinks with added sugar. If they answered yes to either, they were asked to report the number of foods with added sugar in 4 categories (cookies, cake, pie, and donuts; candy; ice cream and frozen treats; and other sugary foods), as well as the number of drinks with added sugar in 4 categories (regular soda pop, juice drinks, coffee shop drink and frozen treats, and other sugary drinks). These EMA survey items, along with dozens of others, were developed for the purposes of our study and were tested in a pilot study [37]. All EMA surveys were designed to be brief (1‐2 min in duration), and those including dietary intake items did not ask participants to specify sizes or types of added sugar items consumed.

Upon enrollment, we collected data on maternal age, education, race, ethnicity, employment status, marital status, and annual household income. We dichotomized some demographic variables for descriptive statistics: age as ≤30 years and >30 years (based on the sample median), employment status as working (part-time or full-time) versus not working (unemployed or on disability), and annual household income as ≤US $50,000 per year versus >US $50,000 per year (based on the sample median). In this study, we also examined EMA completion between participants using their own phone and those using a smartphone provided by PMOMS. Study staff measured participant height at the first clinical trial visit, and participants self-reported their prepregnancy weight at the second clinical trial visit. We calculated prepregnancy BMI by dividing participants’ weight in kilograms by their height in meters squared and categorized participants as normal/healthy (BMI 18.0‐24.9) and overweight (BMI≥25.0) [38].

### Data Analysis

We determined our final sample for this secondary analysis (N=310) after excluding participants only if they were determined to be ineligible for PMOMS (n=3) at the study enrollment phase because they withdrew from either PMOMS or GDM^2^ (parent study). We report frequencies and percentages for all demographic and anthropometric variables.

Completion rates were calculated for all participants included in the analytic sample by dividing the number of responses to dietary intake questions by the number of EOD surveys delivered during both pregnancy and postpartum periods. Given the sampling design described above, we only considered EOD surveys with the dietary intake questions in these completion calculations. We then stratified the study sample across demographic and clinical measures, describing average completion rates for each pair. In addition to the subgroups described above (by age, employment status upon study enrollment, household income), we examined average completion rates for participants by prepregnancy BMI (healthy: 18.0‐24.9 vs overweight: ≥25.0), study phone usage, and race (Black vs White). Lastly, we looked at average completion rates across combined sample strata (eg, by race and age). We focused specifically on Black/African American and White participants in describing completion rates in subgroups due to sample size limitations in other racial groups.

## Results

Table 1 provides an overall description of the analytic sample (N=310), which attempted a total of 36,687 EOD surveys that included dietary intake items, with a mean of 121.5 (SD 70.7) EOD surveys attempted per person (median 2128.0, IQR 60.3-185.8) and a range of 1.0‐243.0 EOD surveys attempted per participant over the study period. Only 3 participants were excluded from these analyses due to being deemed ineligible to participate in PMOMS after enrolling in the study (eg, withdrew from parent study).

**Table 1.** Descriptive statistics of participant demographics and average end of day completion rates in the analytic sample (N=310).

	Sample, n (%)
Using study phone	87 (28.1)
≤30 y old	172 (55.5)
Healthy prepregnancy BMI (18.5‐24.9)	81 (26.1)
Employment	
Full-time	172 (55.5)
Part-time	46 (14.8)
Disability	5 (1.6)
Unemployed/not working	87 (28.1)
Annual household income (US $)	
<20,000	77 (24.8)
21,000‐30,000	41 (13.2)
31,000‐40,000	17 (5.5)
41,000‐50,000	19 (6.1)
51,000‐60,000	22 (7.1)
61,000‐70,000	12 (3.9)
71,000‐80,000	19 (6.1)
>81,000	103 (33.2)
Education	
<High school	16 (5.2)
High school (or equivalent)	57 (18.4)
Some college	65 (21.0)
College degree	82 (26.5)
Master'ss degree	55 (17.7)
Doctoral degree or higher	35 (11.3)
Marital status	
Single (never married)	122 (39.4)
Separated/divorced	11 (3.6)
Married	175 (56.5)
Other	2 (1.0)
Hispanic/Latina	18 (5.8)
Race	
Asian	12 (3.9)
NHOPI^a^	1 (0.3)
Black/African American	87 (28.1)
White	187 (60.3)
More than one race	11 (3.6)
Other	10 (3.2)

a NHOPI: Native Hawaiian or other Pacific Islander.

Compared to the overall demographics of the county where this research was conducted, participants in the present analytic sample are more likely to be Black, Hispanic, or multiracial; be in the labor force; have higher educational attainment; and earn less income annually [39].

Table 2 shows the average completion rates of dietary intake items during the pregnancy (third trimester) and postpartum periods (up to 12 mo after giving birth), as well as across sample strata (eg, study phone vs own phone). We note that values are missing for 6 (1.9%) participants during pregnancy and 11 (3.5%) participants post partum, due to no EOD surveys being delivered to these participants during that time. Rate calculations for the 4 dietary intake items in EOD surveys did not vary between the items, so the completion rates for just 1 item (meals) are presented as proxy rates for overall completion of these items.

**Table 2.** Average completion rates for dietary items during pregnancy and post partum in the overall cohort (N=310), as well as by subgroups and combined strata.

	Average completion rate during pregnancy			Average completion rate post partum		
	Percent, mean (SD)		Sample, n	Percent, mean (SD)		Sample, n
Overall	52.4 (27.8)		299	59.1 (22.0)		304
Study phone	46.7 (29.2)		86	59.2 (20.7)		85
Own phone	54.7 (26.9)		213	59.0 (22.5)		219
BMI<25.0	49.2 (27.5)		77	56.7 (23.6)		80
BMI≥25.0	53.5 (27.8)		222	59.9 (21.4)		224
≤30 y	44.0 (28.6)		163	54.5 (24.2)		169
>30 y	62.5 (23.2)		136	64.9 (17.3)		135
Black/AA^a^	37.3 (27.6)		85	51.3 (24.7)		85
White	58.5 (25.6)		179	62.8 (19.8)		183
Working	43.2 (27.9)		88	59.4 (22.0)		214
Not working	56.3 (26.8)		211	58.4 (22.2)		90
≤US $50,000/y	41.9 (28.4)		148	55.1 (23.5)		150
>US $50,000/y	62.7 (23.0)		151	63.0 (19.7)		154
Black/AA+another variable						
Study phone	40.9 (28.2)		46	57.3 (22.2)		44
Own phone	33.1 (26.7)		39	44.9 (25.9)		41
BMI<25.0	31.1 (24.8)		24	45.9 (25.2)		24
BMI≥25.0	39.8 (28.5)		61	53.4 (24.4)		61
≤30 y	34.3 (27.7)		65	50.5 (25.7)		65
>30 y	47.2 (25.6)		20	53.9 (21.8)		20
Working	39.3 (29.9)		45	46.9 (25.1)		47
Not working	35.2 (25.1)		40	56.8 (23.5)		38
≤US $50,000/y	37.1 (27.7)		79	51.0 (25.3)		79
>US $50,000/y	41.0 (28.9)		6	55.2 (15.6)		6
White+another variable						
Study phone	52.3 (29.3)		28	58.5 (20.0)		29
Own phone	59.6 (24.7)		151	63.6 (19.8)		154
BMI<25.0	56.6 (24.2)		42	60.3 (23.0)		45
BMI≥25.0	59.0 (26.0)		137	63.6 (18.7)		138
≤30 y	51.3 (27.6)		80	57.6 (23.4)		85
>30 y	64.3 (22.3)		99	67.2 (14.8)		98
Working	60.5 (24.6)		142	63.3 (19.8)		143
Not working	50.5 (28.0)		37	60.8 (20.3)		40
≤US $50,000/*y*	46.1 (28.5)		53	60.4 (20.8)		54
>US $50,000/y	63.7 (22.3)		126	63.7 (19.4)		129

a AA: African American.

Overall, participants had higher average completion rates for dietary intake items during the postpartum period (59.1%, SD 22.0%) compared to the pregnancy period (52.4%, SD 27.8%). Looking at subgroup strata, participants using a study phone had lower completion rates on average (46.7%, SD 29.2%) compared to those using their own phone (54.7%, SD 26.9%) during pregnancy, but this difference was nearly eliminated after giving birth (59.2%, SD 20.7% vs 59.0%, SD 22.5%, respectively). Across both pregnancy and postpartum periods, participants who had an overweight BMI before pregnancy (>24.9), were older (>30 years), self-identified as White, were working, or earned a higher income (>US $50,000 annually) had higher average completion rates of dietary intake items.

We also examined completion rates within combined strata, which consisted of race (Black or White) and another variable (eg, phone use), to have a better understanding of variation in responding to EMA surveys within racial groups (Table 2). We observed increased average completion rates from pregnancy to post partum across all strata of Black participants, with the largest increases found for those not working at the time of study enrollment (61%), those with a healthy prepregnancy BMI (48%), and those 30 years old or younger (47%). Black participants using the study phone had considerably higher completion rates on average compared to those using their own phone in both the pregnancy and postpartum periods. During pregnancy, Black participants who were not working at the time of study enrollment (eg, unemployed, on disability) had lower average completion rates (35.2%) compared to those who were working (39.3%), but this was reversed in the postpartum period (56.8% vs 46.9%, respectively).

Similarly, we observed increased average completion rates from pregnancy to post partum across all strata of White participants, although with considerably less variation between time periods. The largest increases were found for White participants earning more than US $50,000 annually (31%) and those who were not working at the time of study enrollment (20%).

**Figure 1.**
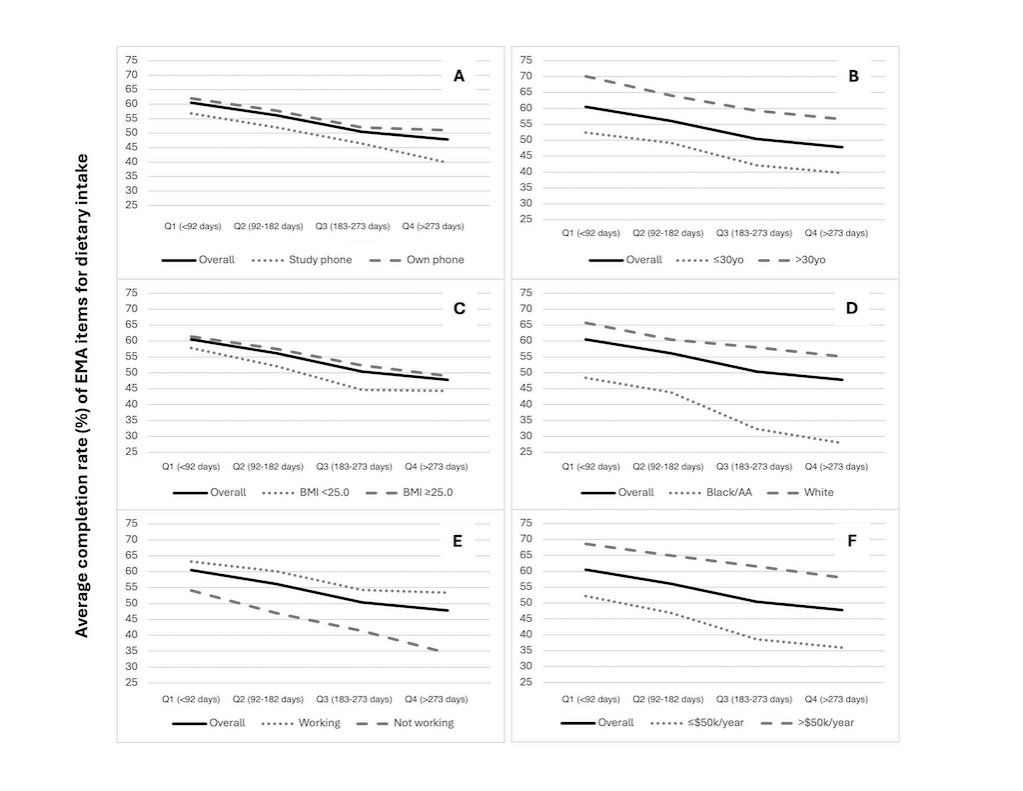
Line graphs depicting average completion rates (%) of EMA items for dietary intake across four postpartum quarters (~91 d) for overall sample, compared against individual characteristics: study phone usage (A); age at study enrollment (B); prepregnancy BMI (C); self-reported race (D); employment status at study enrollment (E); and annual household income (F). AA: African American; EMA: ecological momentary assessment.

Lastly, given the length of the postpartum period (12 mo), we reported completion rates for each postpartum quarter (approximately 3 mo or 91 d) in the overall sample and across subgroup strata (Figure 1). With very few exceptions, completion rates generally decreased over time during the postpartum period, with the lowest rates for all subgroups in the fourth quarter (>273 d after giving birth). We saw similar declining trends in average completion rates for the same combined strata across postpartum quarters (Table S1, Multimedia Appendix 1).

## Discussion

### Principal Findings

Our secondary study is innovative in its examination of overall completion rates for EMA surveys of dietary behavior in a longitudinal, childbearing cohort (~15 mo, from late pregnancy to 1 y post partum), as well as across individual characteristics. Overall, the analytic sample (N=310) had average completion rates of 52.4% during pregnancy (approximately 3 mo) and 59.1% during the first postpartum year (12 mo). This relationship generally held true across subgroups, with average completion rates increasing during the postpartum period, compared to the pregnancy period.

The average completion rates examined here are lower than ideal for EMA research, where at least 80% is often cited as the minimum threshold for “adequate” levels of compliance [40]. Most dietary behavior studies using EMA report adherence rates close to this level or higher [19]. Even so, very few of these studies employed EMA for more than a month, with many of them having study durations of just 1 week [19,41,42]. In our secondary analysis, we observed average completion rates over periods of about 3 months, potentially obscuring higher adherence during shorter intervals. Furthermore, the compensation structure for PMOMS participants was based on monthly rates of EMA adherence, rather than individual survey completion, which may have influenced participant responses.

Our findings partially contradict previous research that demonstrates reduced adherence to EMA protocols over time [19,20,43]. We observed an increase in average completion rates from pregnancy to post partum for all strata examined, potentially attributable to added availability for responding to EMA prompts (ie, maternity leave), reduced stress related to pregnancy symptoms, or increased interest in the study’s objectives regarding postpartum health.

Even so, during the postpartum period, we observed a gradual decline in survey completion across the cohort (Table S1, Multimedia Appendix 1), in agreement with previous literature. We interpret these reductions to survey burden, as well as changing parental obligations, which may impact some populations more than others. In the last postpartum quarter (>273 d after giving birth), our overall cohort had an average completion rate of 47.8%. It is also important to highlight previous evidence demonstrating increased EMA adherence in the morning [20,43,44], whereas the dietary intake items being examined here appeared on the EOD survey.

We observed considerable disparities between subgroups in average completion rates, some of which provide insights into how racial and socioeconomic privilege may impact participation in research applying EMA. Participants who were >30 years old, self-identified as White, and earned >US $50,000 annually had higher completion rates than their counterparts across both prenatal and postpartum periods. Although research examining demographic differences in EMA adherence is limited, a 9-week smoking cessation study found greater declines in adherence for participants who were younger, working full-time, and not White [44]. These differences in EMA completion rates may be attributable to the socioeconomic advantages associated with privileged groups, such as increased access to resources (eg, employment, childcare) and reduced exposure to stressors (eg, food insecurity, housing instability). Chronic exposure to stressors, particularly related to caregiving, may exacerbate EMA burden for participants, even from one day to the next [42].

During the first postpartum year, average completion rates increased by 38%, 35%, 32%, and 24% for participants who self-identified as Black, reported not working (upon study enrollment), had lower income (≤US $50,000 annually), and were 30 years old or younger, respectively. These increases were more considerable than those observed among the privileged subgroups, which ranged from 0.5% to 7%. Beyond the experience of survey burden for participants, these findings suggest that the prenatal period carries additional burdens for childbearing people from racially and socioeconomically oppressed groups, potentially inhibiting their participation in research.

Examining combined strata here provided additional insights that would not have been afforded to us if we had ceased the analysis at single-identity categories. Black participants using the study phone had average completion rates approximately 20% higher than those using their own phone, across the study period, contrasting with results from both the overall study cohort and strata of White participants. We consider this a novel finding in EMA research, where study designs typically leverage the use of the participant’s current mobile device, potentially excluding those who lack compatible devices or access to quality phone or internet service [41].

### Strengths and Limitations

While this study offers novel, scientific insights into demographic and socioeconomic factors associated with completion of EMA surveys regarding food intake in a longitudinal, childbearing cohort, it is not without its limitations. First, PMOMS is an ancillary study to a randomized clinical trial, which had specific exclusionary criteria (eg, history of diabetes diagnosis) that may have ultimately biased EMA survey responses within the cohort. Second, examining combined strata (eg, race and income) resulted in some small samples, making our study underpowered to conduct comparative analyses or report statistical significance. For this reason, we decided to provide descriptive results only. Future investigations of variation in EMA participation across sociodemographic factors would benefit from a larger, representative sample to apply comparative methods (ie, linear regression). Next, we conducted our assessment of employment status upon study enrollment (during late pregnancy). We asked about employment status in an exit survey within an administrative supplement of the study, meaning only a subset of the population had employment information at the conclusion of the study. Lastly, we would also like to qualify that our study findings do not include information about any technical issues (eg, device malfunction, survey delivery failure), which would likely impact participants’ EMA completion. Although the PMOMS team documented these issues throughout the study period to assist participants and troubleshoot, these data were not prepared to be included in these analyses.

### Public Health Implications

The findings presented here are novel and valuable to perinatal health research, particularly that which applies EMA or telehealth technologies to collect data in diverse, representative cohorts for longer periods (eg, several weeks, months). Although PMOMS is considerably longer than most EMA studies, here we demonstrated relatively stable engagement (average completion rate of 59%) in a childbearing population over the course of 15 months. Given evidence linking contextual factors (eg, stress) to parental behavior, including dietary behaviors [41], EMA may serve as a better methodological tool in maternal health research compared to more burdensome dietary recalls.

Our descriptive analysis of EMA completion rates in a childbearing cohort has important implications for future research and intervention designs. PMOMS remains unique in its application of EMA over more than 12 months, though there is room for improving completion rates in longer EMA studies. Immediately actionable strategies include reducing the number of EMA surveys per day or week and increasing opportunities for compensation among participants. Another approach would be increasing participant engagement through an online dashboard where they can see their own response data, as well as aggregate data for the entire cohort.

We recommend that investigators consider the intersections between race and other factors (eg, employment) in relation to study participation and develop strategies to improve EMA completion (eg, study-provided smartphone) among participants who are likely to face more socioeconomic burdens.

## Supplementary material

10.2196/67081 Multimedia Appendix 1 Average completion rates (%) for dietary intake items by postpartum quarter (~91 d) across combined strata (race+another variable).

## Abbreviations

BOD: beginning of day
EMA: ecological momentary assessment
EOD: end of day
GDM^2^: Comparison of Two Screening Strategies for Gestational Diabetes
PMOMS: Postpartum Mothers Mobile Study
