# Supplementary material for: Completion Rates for Ecological Momentary Assessments of Food Intake During Pregnancy and Post Partum: Descriptive Study
Source: JMIR Hum Factors. 2025 Oct 8;12:e67081. doi: 10.2196/67081 (PMC12507379; doi:10.2196/67081)
Supplement: Multimedia Appendix 1 [file humanfactors-v12-e67081-s001.docx]

**Supplemental Table.** Average completion rates (%) for dietary intake items by postpartum quarter (~91 days) across combined strata (race + another variable).

|  | **Postpartum** | | | |
| --- | --- | --- | --- | --- |
|  | Q1 (<92 days) | Q2 (92-182 days) | Q3 (183-273 days) | Q4 (>273 days) |
| **Black/AA + Study phone** | 53.7 | 47.6 | 37.2 | 31.9 |
| **Black/AA + Own phone** | 42.1 | 38.8 | 26.8 | 23.7 |
| **White + Study phone** | 60.0 | 56.1 | 57.9 | 45.4 |
| **White + Own phone** | 66.7 | 61.3 | 57.9 | 56.9 |
|  |  |  |  |  |
| **Black/AA + BMI <25.0** | 43.4 | 36.3 | 23.6 | 23.6 |
| **Black/AA + BMI ≥25.0** | 50.3 | 46.2 | 35.6 | 29.6 |
| **White + BMI <25.0** | 64.3 | 57.0 | 53.7 | 52.3 |
| **White + BMI ≥25.0** | 66.1 | 61.5 | 59.2 | 55.8 |
|  |  |  |  |  |
| **Black/AA + ≤30yo** | 44.8 | 40.7 | 29.8 | 26.4 |
| **Black/AA + >30yo** | 59.9 | 51.9 | 39.2 | 33.1 |
| **White + ≤30yo** | 58.3 | 54.6 | 51.6 | 50.1 |
| **White + >30yo** | 71.7 | 65.1 | 62.6 | 58.5 |
|  |  |  |  |  |
| **Black/AA + Working** | 48.4 | 47.5 | 36.5 | 35.4 |
| **Black/AA + Not working** | 48.2 | 39.1 | 27.8 | 19.5 |
| **White + Working** | 66.8 | 62.2 | 59.1 | 57.8 |
| **White + Not working** | 61.3 | 54.1 | 54.0 | 44.9 |
|  |  |  |  |  |
| **Black/AA + ≤$50k/yr** | 47.9 | 43.9 | 32.1 | 27.3 |
| **Black/AA + >$50k/yr** | 54.6 | 37.2 | 34.8 | 35.9 |
| **White + ≤$50k/yr** | 57.0 | 48.8 | 45.8 | 45.1 |
| **White + >$50k/yr** | 69.3 | 65.3 | 62.6 | 58.4 |
